# Systemic dizocilpine (MK-801) facilitates performance in opposition to response bias

**DOI:** 10.1186/1744-9081-3-48

**Published:** 2007-09-19

**Authors:** Regan G Wisnewski, Johan Lauwereyns

**Affiliations:** 1 School of Psychology, Victoria University of Wellington, Wellington, New Zealand

## Abstract

Previous research has established that dopamine signals are crucial in orienting behavior to reward. Less is known, however, about the psychopharmacology of task performance under small-reward conditions as compared to large-reward conditions. The current study examined the effects of the noncompetitive *N*-methyl-D-aspartate (NMDA)-receptor antagonist dizocilpine (MK-801) on reaction time (RT) in a nose-poke task with rats completing an asymmetric reward schedule. In all trials, the rats were required to poke their nose in either the left or the right peripheral hole immediately adjacent to the centre hole when the corresponding light was illuminated. Depending on the stimulus-reward mapping, however, one position was associated with a large reward, while the alternative position was associated with a small reward. Correct performance was required in every trial; if the rat did not make a correct response within 20 *s*, the trial was aborted, and the same stimulus was presented again on the next trial. In this way, the rat was forced to perform the same visuo-spatial discrimination task under different reward conditions. Reaction times (*ms*) were faster for large-reward trials than for small-reward trials, replicating previous findings. At a dosage of MK-801 (0.04 *mg/kg*), there was no significant influence of on RT in large-reward trials. In contrast, the same dosage of MK-801 in small-reward trials produced a decrease in RT as compared to the control condition, implying an improvement of performance. Below 0.04 *mg/kg *of MK-801, a steady decrease of RT in small-trials was seen as a function of dosage. Above 0.04 *mg/kg *of MK-801, the majority of rats failed to perform the task at all, whereas the rats that did manage to perform the criterion of 80 correct trials in a session showed no difference in RT between large- and small-reward trials. These data indicate that the systemic administration of a relatively small dosage of MK-801 facilitates performance when reward is small. It is suggested that the facilitation may be due to the reinforcement of mechanisms that work in opposition to response bias. As a corollary, the study provides a useful paradigm to study the voluntary control of unavoidable action.

## Findings

Responses are faster and more accurate with a large reward than with a small reward [1]. It is thought that predictive signals of dopamine neurons are crucial in orienting behavior to reward [2]. For instance, dopamine input to dorsal striatum may induce a response bias, with a high *a priori *likelihood to choose a response associated with a large reward [3,4]. However, many situations impose a mandatory requirement in favor of a less-desirable option. Recent evidence showed that thalamic neurons in macaque monkeys reacted with a burst of activity after stimulus presentation, but only in small-reward trials, not in large-reward trials [5]. This neural activity may counteract the response bias and reinforce the less attractive response [6]. Given that these thalamic neurons are not a major target of dopaminergic projection, it is likely that the counteractive mechanism is not under direct control of dopamine.

By using an asymmetric reward paradigm, we are able to investigate response bias and its opponent action in different trials of the same task. In this paradigm, subjects are always required to perform a spatial response to a visual cue, but receive either a large or a small reward for a correct response, depending on the position-reward association. Here, subjects typically develop a response bias in the direction associated with a large reward [7]. In large-reward trials, this response bias actually matches with the required response, and so any process observed in large-reward trials likely pertains to the neural mechanism of response bias. In small-reward trials, however, there is a mismatch between response bias and the required response, calling for the counteractive mechanism to intervene. Any process that is uniquely linked to small-reward trials, then, would most likely reflect this counteractive mechanism.

In the present study, we were particularly interested in selective influences on behavior in small-reward trials. Recently, by DNA targeting of the dopamine D2 receptor protein in rhinal cortex, a selective improvement of monkeys' performance was observed in trials that were not associated with immediate reward, whereas performance was unaffected in trials that did lead to immediate reward [8]. No such influence was obtained with a DNA construct that decreased the amount of ligand binding to NMDA receptors in rhinal cortex. Inspired by the findings in thalamus [5], we explored the possibility that the null result of NMDA antagonism [8] was peculiar to rhinal cortex. We examined whether the NMDA-receptor antagonist MK-801, systemically administered, might selectively improve behavior in small-reward trials using an asymmetric reward paradigm with nose poke responses. We opted for nose pokes as they are natural responses for rats, and provide a very sensitive dependent measure in the form of reaction time [7].

## Methods

### Subjects

Subjects were 10 male Sprague-Dawley rats, weighing 190 – 230 *gm *(85% of their free-feeding weight). All experimental protocols were approved by the Victoria University of Wellington Animal Ethics Committee.

### Behavioral Apparatus

Two 9-hole boxes (MED-NP9L-B1; MED Associates, St Albans, VT) were used to conduct the experiments. Both boxes contained an arc of 9 contiguous apertures set into the curved front wall. Each aperture was 2.5 *cm *× 2.5 *cm *square and 2.2 *cm *deep. Light-emitting diodes at the rear of each hole were turned on and off automatically to provide visual cues specific to each hole. Vertical infrared detectors at the front of each nose-poke hole allowed the recording of response latencies and locations. A 0.1 *ml *reinforcer (20% sucrose solution) was delivered via a metal dipper centered in the rear wall.

### Behavioral paradigm

**Figure 1:**
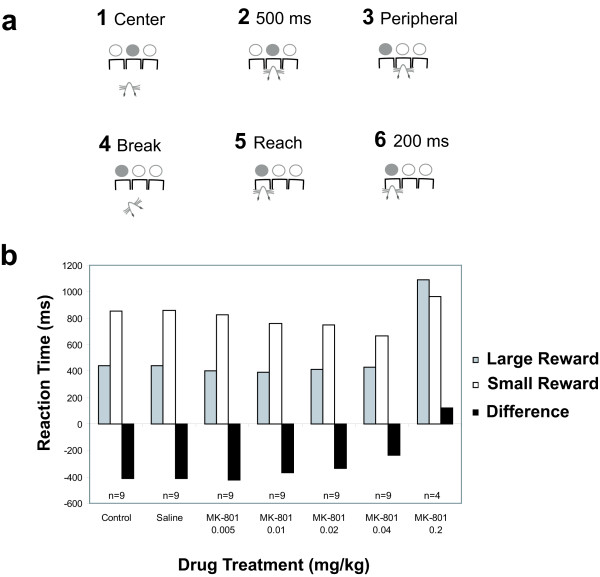
**a**) Schematic representation of the sequence of events in a single trial. The trial started with the onset of the center LED. The rat was required to poke its nose in the corresponding hole, and stay in this position for 500 *ms*. At this time, the peripheral stimulus was presented and the center LED was extinguished. The trial ended when the rat poked its nose and stayed for 200 *ms *in the hole adjacent to the center hole corresponding with the illuminated light. *RT *(*ms*) was defined as the time duration between onset of peripheral stimulation and the moment when the rat poked its nose in the correct response hole, provided that it remained there for 200 *ms*. **b**) Psychopharmacological data with systemic administration of MK-801. Data from nine rats that were able to reach the performance criterion of 80 correct trials for dosage levels of 0.04 *mg/kg *or less. At the highest dosage level, only four rats were able to complete more than 80 trials.

Sessions were conducted daily, for a maximum of 200 trials or until 40 *min *had elapsed. The behavioral paradigm comprised the following events (*see *Figure 1a). A trial started when the center hole light was illuminated, signaling the rat to make a nose-poke response and sustain it for 500 *ms*. Once a nose-poke response had been sustained for 500 *ms *in the center hole, the light was extinguished. Instantaneously, and unpredictably, either the left or right light in the hole adjacent to the center hole was illuminated. The rat had to poke its nose in the illuminated hole for at least 200 *ms*. If the rat failed to do so within 20 *s*, an identical trial was presented after 30 *s *(*i.e.*, correction procedure).

To investigate the influence of incentive, an asymmetrical reward schedule was used. Rats were permanently assigned to a particular position-reward mapping. For five of 10 rats, the *left*-to-centre peripheral nose poke was always worth 0.3 *ml *of reinforcer (3 × 0.1 *ml *dipper: large reward condition) and the *right*-to-center peripheral nose poke was always worth 0.1 *ml *of reinforcer (1 × 0.1 *ml *dipper: small reward condition). For the other five rats, the reward schedule was reversed. Thus, the rat acquired fixed position-reward associations, but could not predict where the target would appear in any trial.

### Pharmacological Procedure

All rats received different dosages of MK-801 (0.005, 0.01, 0.02, 0.04 and 0.2 *mg/kg*) in separate daily sessions, conducted in counterbalanced order with three-day intervals between experimental sessions. MK-801 was dissolved to the appropriate dose in 0.9% saline solution prior to delivery and was administered via intraperitoneal injection, 30 *min *prior to the start of a testing session.

## Results

*Reaction time (ms) *was recorded as the latency of nose entry in the peripheral hole after onset of the peripheral light stimulus. For MK-801 dosages of up to 0.04 *mg/kg*, the data from the initial experiment (*see *Figure 1b) showed a dose-dependent decrease of RT in small-reward trials, whereas RT appeared unaffected in large-reward trials. Above 0.04 *mg/kg *of MK-801, the majority of rats failed to perform the task at all, whereas the four rats that did manage to perform the criterion of 80 correct trials in a session showed no difference in RT between large- and small-reward trials. To evaluate the reduction effect on RT statistically, we collected more data with the dosage of 0.04 *mg/kg *MK-801. Each rat completed two experimental sessions under MK-801 (0.04 *mg/kg*) treatment interspersed between nine control sessions without pharmacological treatment.

Only data from rats that consistently performed more than 80 correct trials per session were included. Based on this criterion, the data from two rats were discarded. A repeated measures ANOVA on RT showed that there was a highly significant effect of the factor Reward, F(1,7) = 161.83, MSE = 4996, p < .001, with faster RTs in the direction associated with a large reward (340 *ms*) than in the direction associated with a small reward (658 *ms*). There was also a very reliable main effect of the factor Treatment, F(1,7) = 7.63, MSE = 2096, p < .05, with faster RTs for the MK-801 condition (477 *ms*) than for the control condition (521 *ms*). Finally, there was also a highly significant interaction between Reward and Treatment, F(1,7) = 13.19, MSE = 1267, p < .01. In large-reward trials, pairwise comparisons revealed no significant differences in the speed of overall RTs between MK-801 and control conditions. However, in small-reward trials, a significant difference was observed in the speed of overall RTs between MK-801 and control conditions, t(7) = 3.295, p < 0.05: With MK-801 (613 ms) the responses were 90 ms faster than in the control condition (703 ms).

## Discussion

Using a rat nose-poke paradigm with an asymmetric reward schedule, we observed faster RTs for large-reward trials than for small-reward trials. Blocking the action of glutamate by antagonizing NMDA-receptors with a relatively small dosage of MK-801 (0.04 *mg/kg*) led to a speeding up of responses that are otherwise performed rather sluggishly. Specifically, in large-reward trials there was no significant influence of MK-801 on RT compared to the control condition, whereas in small-reward trials MK-801 produced a decrease in RT compared to the control condition.

Our results concur with previous research findings indicating that NMDA-receptors are critically involved in the guidance of instrumental behavior (RT) as a function of reward magnitude [9,10]. At the same time, the current findings go one step further, indicating that antagonizing NMDA-receptors produces an improvement of performance in small-reward trials. As such, these findings shed new light on the null effect from an NMDA antagonist in rhinal cortex [8]. Future research will need to determine which neural structures are responsible for the dizocilpine-dependent reduction of reaction time in small-reward trials.

In line with the proposal that different types of trials reflect separate underlying mechanisms in the present asymmetric reward paradigm, we suggest that the observed effect of MK-801 is due to selective enhancement of the neural mechanism that counteracts response bias. For instance, it may be that thalamic neurons, which encode the counteractive mechanism to response bias, become more easily activated, or succeed more quickly in generating an opponent action, when glutamatergic influences (e.g., from cortex on dorsal striatum) are lifted that would otherwise lead to perseveration of response bias. As such, the present results attest to the usefulness of the current paradigm to study the voluntary control of actions that have little immediate appeal or direct incentive, but are unavoidable in the pursuit of rewards in future trials.

## Abbreviations

ANOVA - Analysis of variance;

MK-801 - Dizocilpine;

MSE - Mean squares of error;

NMDA - *N*-methyl-D-aspartate;

RT - Reaction time.

## Competing interests

The author(s) declare that they have no competing interests.

## Authors' contributions

RW carried out the data collection, participated in its design and coordination and drafted the manuscript. JL conceived of the study, and participated in its design and coordination and helped to draft the manuscript. Both authors read and approved the final manuscript.
